# Challenges and perspectives in the application of isothermal DNA amplification methods for food and water analysis

**DOI:** 10.1007/s00216-018-1553-1

**Published:** 2019-01-08

**Authors:** Roland Martzy, Claudia Kolm, Rudolf Krska, Robert L. Mach, Andreas H. Farnleitner, Georg H. Reischer

**Affiliations:** 1 TU Wien, Institute of Chemical, Environmental & Bioscience Engineering, Molecular Diagnostics Group, Department of Agrobiotechnology (IFA-Tulln), 3430 Tulln, Austria; 2 ICC Interuniversity Cooperation Centre Water & Health, Vienna, Austria; 3 University of Natural Resources and Life Sciences, Vienna (BOKU), Department of Agrobiotechnology (IFA-Tulln), Konrad-Lorenz-Str. 20, 3430 Tulln, Austria; 4 Queen’s University Belfast, Institute for Global Food Security, School of Biological Sciences, Belfast, Northern Ireland BT71NN UK; 5 TU Wien, Institute of Chemical, Environmental & Bioscience Engineering, Research Area Biochemical Technology, Research Group of Synthetic Biology and Molecular Biotechnology, 1060 Vienna, Austria; 6 Karl Landsteiner University of Health Sciences, Research Unit Water Quality and Health, 3500 Krems, Austria; 7 TU Wien, Institute of Chemical, Environmental & Bioscience Engineering, Research Area Biochemical Technology, Research Group of Environmental Microbiology and Molecular Diagnostics, 1060 Vienna, Austria

**Keywords:** Molecular diagnostics, Isothermal DNA amplification, Point-of-care testing, Low-resource settings, DNA extraction, Ionic liquids

## Abstract

Molecular diagnostic tools in the field of food and water quality analysis are becoming increasingly widespread. Usually, based on DNA amplification techniques such as polymerase chain reaction (PCR), these methods are highly sensitive and versatile but require well-equipped laboratories and trained personnel. To reduce analysis time and avoid expensive equipment, isothermal DNA amplification methods for detecting various target organisms have been developed. However, to make molecular diagnostics suitable for low-resource settings and in-field applications, it is crucial to continuously adapt the working steps associated with DNA amplification, namely sample preparation, DNA extraction, and visualization of the results. Many novel approaches have been evaluated in recent years to tackle these challenges, e.g., the use of ionic liquids for the rapid isolation of nucleic acids from organisms relevant for food and water analysis or the integration of entire analytical workflows on microfluidic chips. In any event, the future of applications in the field of isothermal amplification will probably lie in ready-to-use cartridges combined with affordable handheld devices for on-site analysis. This trend article aims to make prospective users more familiar with this technology and its potential for moving molecular diagnostics from the laboratory to the field.

**Figure:**
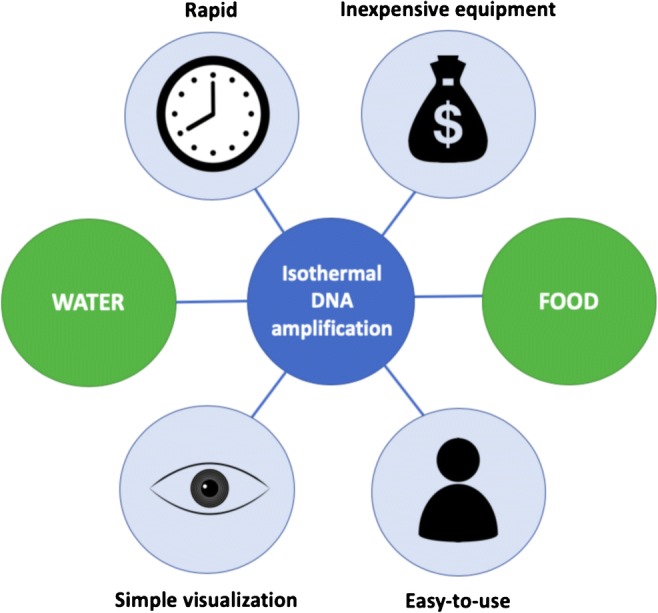
Graphical abstract

Graphical abstract

## Introduction

In this trend article, the state-of-the-art and recent advances of isothermal DNA amplification methods for food and water analysis are presented by providing a broad overview on promising new developments and existing challenges. Rather than reviewing this field in its completeness, or listing assays and their performance characteristics, our aim is to point the reader towards some of the most recent developments and papers on the respective topics. In doing so, we not only focus on the latest scientific research but also give an insight into what has been made commercially available thus far. These aspects are illustrated by a case study on the implementation of an isothermal amplification method for microbiological water quality analysis. Finally, future improvements are proposed that are needed to realize the vision of cost-effective molecular diagnostic tools for field applications.

## Importance of molecular diagnostics for food and water safety

**Fig. 1:**
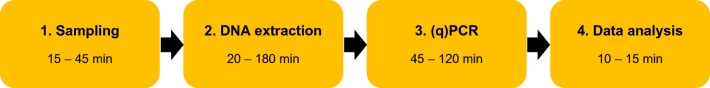
Schematic representation of a molecular diagnostic analysis pipeline. The time required depends on the target analyte, spanning 90 to 360 min for the entire workflow. Steps 2–4 typically require a sophisticated laboratory infrastructure (e.g., cell homogenizer and thermal cycler)

Although the term molecular diagnostics is primarily associated with clinical diagnostics, we explicitly consider molecular methods to analyze food, feed, and water quality as part of this field. Molecular diagnostics include various methods for detecting nucleic acids (RNA and DNA) and proteins, but this article focuses solely on methods for detecting DNA. Thus far, the most commonly used technique for DNA detection is (quantitative) polymerase chain reaction (q)PCR, which is an in vitro amplification method that produces up to a billion-fold copies of a particular target DNA sequence through an enzymatic reaction. With (q)PCR, it is possible to detect any living organism that has left traces of its genetic information in a sample (see Fig. 1 for a schematic representation of a typical analysis workflow). To increase the experimental transparency of such molecular diagnostic tests, the Minimum Information for Publication of Quantitative Real-Time PCR Experiments (MIQE) guidelines were defined in 2009 [1]. These guidelines aim to ensure the reliability of the results by describing the minimum information required to evaluate newly developed qPCR assays, thereby promoting consistency among different laboratories. For these reasons, qPCR has become an essential tool to detect genetic modifications in commodities intended for trade or to check for the presence of allergenic plants or human pathogens in food [2, 3]. Antimicrobial resistance, which is of great public health importance in the present and future, can also be traced by screening methods based on (q)PCR [4]. Furthermore, DNA-based methods can be used to verify the authenticity of meat or perform routine quality controls in the water supply chain. To this end, various regulations have been introduced worldwide to set certain standards—e.g., Regulation (EC) No 1830/2003 concerning the traceability and labelling of genetically modified organisms in the European Union [5].

## Future demands on molecular diagnostics

In an ideal conception, future molecular diagnostic methods are performed using inexpensive and robust equipment that can be easily taken out into the field to the desired analysis site (often denoted as “point-of-care” in clinical diagnostics). To allow a broad application even in resource-limited settings (e.g., developing countries), reagents or disposables should also be highly affordable and require no special disposal procedures. In terms of analysis time, these methods should provide a result at least within 30 min to allow a quick response to potentially unforeseen or undesirable outcomes. Finally, the methods should also be readily applicable by non-molecular biology trained personnel, indicating that the number of pipetting steps is minimized, and handling is kept intuitive to avoid sources of error. These requirements were, for the first time, officially summarized by the World Health Organization (WHO) in their ASSURED guidelines for point-of-care testing (**A**ffordable, **S**ensitive, **S**pecific, **U**ser-friendly, **R**obust and rapid, **E**quipment-free, **D**eliverable to those who need them) [6]. Although the criteria have been developed for molecular diagnostics of diseases, we believe that they should also be used as a future benchmark for the analysis of food and water in point-of-care settings.

## Advent of isothermal DNA amplification methods

One of the steps to meet these demanding requirements has been the development of isothermal DNA amplification (IsoAmp) methods over the last two decades. In 2000, Notomi et al. developed the loop-mediated isothermal amplification (LAMP) technique [7], which, today, is the most widely studied and applied isothermal DNA amplification method. Various other methods, all with their own advantages and limitations, followed—e.g., helicase-dependent amplification (HDA), recombinase polymerase amplification (RPA), rolling circle amplification (RCA), and cross-priming amplification (CPA), among many others [8, 9]. Although they are based on different reaction principles, all methods have in common that the amplification of the targeted gene occurs at a constant temperature, thus eliminating the need for sophisticated laboratory equipment such as thermal cyclers. Instead, a heating block or water bath is sufficient to provide the necessary conditions to conducting the analysis. Due to the lack of heating and cooling steps, many of the isothermal methods are faster than (q)PCR methods, depending on the target and particular assay. These properties open completely new application areas—e.g., in basic laboratories that are not equipped for DNA-based analyses. Moreover, isothermal DNA amplification methods can be implemented in resource-limited settings, potentially moving molecular diagnostics from centralized laboratories directly into the field to the sampling site.

**Fig. 2:**
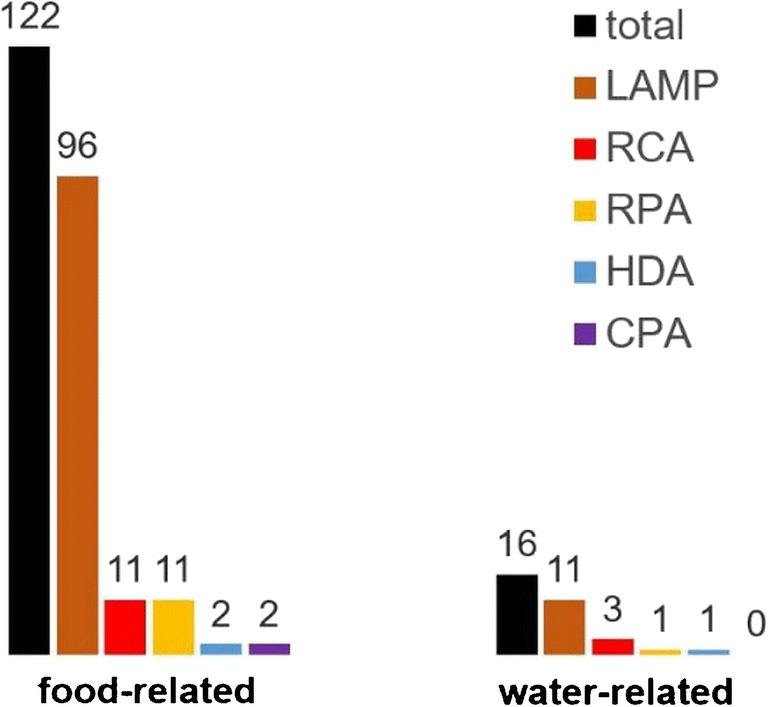
Number of publications (January 2014 to September 30, 2018) describing assays based on the most prominent isothermal amplification methods in food and water analysis (Web of Science Core Collection, September 30, 2018)

Using these techniques, many assays have been published since the first isothermal methods were introduced. The potential applications cover almost every field of research, from clinical to environmental to food and feed diagnostics, spanning the detection of plant and animal species to pathogenic microorganisms. A database search for isothermal amplification methods focussing on the analysis of food and water showed that 78% and 69%, respectively, are based on LAMP (Fig. 2). LAMP is characterized by a high degree of robustness and high specificity and sensitivity and has been shown to be very insensitive to various inhibitory substances. This is attributed to the polymerase used for LAMP reactions (*Bst*I), which proved to be very robust to inhibitory substances and remained sensitive and specific even in the presence of difficult specimens such as urine or stool [10]. This robustness is becoming increasingly important in the analysis of complex matrices found in many types of foods, feeds, or polluted water samples.

## Case study: Towards implementing a field-applicable workflow to detect health-relevant bacteria in water

To illustrate the challenges of implementing isothermal assays for in-field monitoring, we selected a case study addressing the molecular detection of health-relevant bacteria in water. Briefly, when water resources are contaminated with fecal material, potentially co-excreted pathogenic microorganisms pose a hazard to public health. Thus, it is necessary to routinely assess the microbiological quality of water that will be eventually used for drinking, bathing, or other purposes. For decades, microbiological water quality has been investigated by cultivating standard fecal indicator bacteria (SFIB) such as *Escherichia coli* or *Enterococcus* species. Recently, molecular methods based on the detection of genetic markers for SFIB by quantitative polymerase chain reaction (qPCR) have been developed and implemented in the USA [11]. As mentioned above, four major steps are necessary when conducting such an analysis—target enrichment, sample preparation, target quantification, and visualization/data analysis. Following these steps, we will compare the current molecular diagnostic methodology with a visionary, field-applicable workflow based on isothermal DNA amplification. On the one hand, we will tackle the specific challenges involved in implementing a new isothermal method for water quality monitoring. However, in doing so, we will also examine the challenges that still accompany this new technology in food-related applications.

### Step 1: Sampling—target enrichment

Because SFIB might be present in the sample in very low numbers, it is necessary to enrich the bacteria. This is achieved by filtering a certain volume of water (milliliters to several liters) to concentrate the bacteria on a membrane filter. In the laboratory, this can be performed using a vacuum pump and a convenient filtration apparatus; however, when considering low-resource settings or applications directly in the field, this method immediately constitutes a challenge. One possible solution is to use disposable syringes with specially designed attachments containing membranes that can be exchanged after each filtration. Jiang et al. demonstrated that the filter can be subsequently backflushed to recover the cells in a smaller volume (approximately 200 μl) of water or buffer [12]. Another solution is the commercially available Aguaguard sytem, a portable device that is designed to concentrate microorganisms from a water sample on-site and to recover them in a minimum of 1 ml of elution buffer. Although this method might not be economically feasible for developing countries, it is still interesting for applications in remote areas without access to a laboratory infrastructure. However, to our knowledge, there are no studies that directly compare the performance of molecular water quality analyses using different filtration approaches. In any case, existing sampling and sample preparation guidelines such as those for GMO analysis [13], or the ISO standards for water sampling for microbiological analysis [14], should be strictly adhered to. They help avoid sampling errors and make sample collection reproducible and standardized, independent of the subsequently applied molecular diagnostic tools.

### Step 2: Sample preparation—nucleic acid extraction

In general, the extraction of nucleic acids (DNA or RNA) is a crucial step in the molecular diagnostic analysis of food and water, owing to the diverse and complex matrices containing the analyte of interest. Conventionally, DNA extraction is achieved by mechanical, enzymatical, or chemical lysis of the cells containing the analyte, followed by chemical extraction using phenol and chloroform and the subsequent purification of the nucleic acids—e.g., by precipitation. Commercial extraction kits for DNA and RNA use similar principles, although the use of toxic chemicals is mostly avoided. Both standard procedures and commercial kits are highly dependent on laboratory infrastructure (e.g., fume hood, cell homogenizer, centrifuges, vacuum pumps, and disposal facilities for toxic waste). In any case, the extraction usually takes several hours per sample and requires trained personnel. This finding is in direct contrast to the idea of rapid molecular methods that can be applied at the point of interest, rendering these approaches impractical in low-resource settings. This also applies to the case study in which the genetic material from bacterial cells enriched from the filtrated water sample must be isolated and purified prior to the analysis. In addition to sampling itself, sample preparation is the most crucial bottleneck in molecular diagnostics, still hindering the application of subsequent isothermal DNA amplification methods to be applied in the field or in resource-limited settings. This is also true for food-related analyses, especially when extracting nucleic acids from processed foods or complex matrices (e.g., foods with a high fat content). Researchers also attempted to completely avoid nucleic acid isolation and instead apply the sample directly into the isothermal amplification mix. This has been demonstrated for plants using the example of papaya [15] and for the fecal indicator bacteria *E. coli* and *E. faecalis* [16]. The authors concluded that the elevated temperature of 63 °C could lead to increased cell permeability, allowing direct amplification of the target DNA. Depending on the intended application, it might be feasible for qualitative measures when sample size is not an issue. However, this approach remains questionable when the target analyte is only present in trace amounts—e.g., in environmental samples or contaminated food commodities.

To tackle this challenge, novel procedures have been proposed in recent years that use ionic liquids (ILs) for the rapid extraction of DNA from foods and Gram-negative bacteria [17, 18]. Using this approach, the samples are incubated with the ILs for several minutes at a given temperature and can be subsequently used for (isothermal) amplification reactions. For example, our group has recently developed an IL-based method to extract DNA from various types of meat [19]. Biodegradable choline hexanoate in sodium phosphate buffer was used for sample lysis, resulting in a high DNA yield. Stabilization of the extracted DNA by ILs even allowed storing the extracted DNA at room temperature for up to 20 days without significant losses. Only recently, we discovered two hydrophilic ILs capable of lysing Gram-positive and Gram-negative bacteria that are important for the assessment of food or water quality (unpublished data). While ionic liquids will have to be specifically selected and evaluated for each desired target organism and sample matrix, we believe that they represent some of the most promising approaches for rapid and simple DNA extraction to be used in field-applicable diagnostics.

### Step 3: DNA amplification

After isolating the nucleic acids from the sample, the desired target sequence is amplified (i.e., multiplied) using RNA or DNA amplification methods such as reverse transcription (RT)-(q)PCR. In the case of water quality analysis using fecal indicator bacteria, the USEPA recommends using a probe-based qPCR assay targeting a diagnostic fragment of the 23S rRNA gene specific for the relevant indicator—i.e., *Enterococcus* species [11]. However, this analysis requires a qPCR thermal cycler capable of monitoring the amplification procedure in real time. To avoid the necessity of complex equipment, our group developed a LAMP [20] and an HDA assay [21] that target the same DNA sequence and were shown to have comparable specificity and sensitivity as the USEPA assay. While both new methods can be performed entirely on a heating block, the LAMP assay is faster with a reaction time of only 45 min, whereas the HDA assay has a significantly lower detection limit. Several other isothermal amplification assays have been reported that target health-relevant microorganisms in water (e.g., *Escherichia coli* or various pathogenic bacteria). The same applies to a vast number of LAMP assays to detect food pathogens and fungal contaminants [22] or for the quality assessment of meat [23]. Among the four steps covered in this section, this is where most of the work has been done in recent years. Moreover, many isothermal DNA amplification methods targeting important pathogens have already been diligently studied or evaluated, such as LAMP assays to detect *Salmonella* in food and feed by the Food and Drug Administration of the United States [24]. Although the abovementioned MIQE guidelines have been defined primarily for qPCR experiments, it will be of utmost importance to extend them to isothermal DNA amplification assays to establish certain quality standards in the future.

Compared with (q)PCR, isothermal amplification assays are often more complex and challenging in their design due to specific properties of the methods—e.g., the use of six or more primers in LAMP, specific melting temperature of HDA products, or low amplification temperature of 37–42 °C for RPA reactions. Although these characteristics of isothermal amplification methods make it challenging, it has been demonstrated that multiplexing allows simultaneous detection of different target sequences in a single tube reaction [25].

### Step 4: Visualization and data analysis

In the conventional DNA-based detection of SFIB, amplification of the target DNA is detected either by visualization of the reaction product (PCR amplicon) by agarose gel electrophoresis or by real-time monitoring during amplification in a qPCR cycler. The products of LAMP assays are often visualized by the addition of a chemical dye after completion of the reaction [26]. In this procedure, the reaction chamber must be opened to add the chemical dye, severely increasing the risk of carry-over contamination and potentially leading to false-positive results. Work-arounds include introducing restriction endonuclease recognition sites in the primer sequences, allowing the degradation of the DNA after the analysis [27], or the use of wax-encapsulated fluorescent dyes in the reaction mixture that are melted upon completion of the reaction, setting free their content [28]. Alternatively, LAMP can be modified to produce large quantities of white magnesium pyrophosphate precipitate during the reaction. Eiken Chemical Co., Ltd. (Tokyo, Japan) designed the simple Loopamp Realtime Turbidimeter that can monitor the formation of this turbidity. Another detection strategy is based on low-cost, easy-to-use lateral flow assays that are suitable for endpoint analysis of amplification products in low-resource settings [29].

To quantify the DNA concentration in the sample using qPCR, plasmid standards containing the DNA sequence of interest are measured alongside the samples. However, this requires the use of a thermal cycler that allows monitoring of the amplification in real time. Considering our very simple, low-resource case example, it will not be possible to quantify the target analyte when the amplification reaction is conducted on a heating block without any connected optical monitoring device. The addition of chemical dyes after the reaction only allows a qualitative statement regarding whether the targeted DNA was present in the sample. Without access to expensive laboratory equipment, a most probable number (MPN) format or a microchip device with highly replicated/parallelized reaction chambers might be used to quantify the target analyte [16]. As a promising alternative, a standard smartphone with its camera could be used as a detector for the fluorescence of intercalating dyes that bind to the new DNA formed during an (isothermal) amplification reaction in real time and allows a quantification of the target DNA [30].

### Conclusion on the novel workflow to detect health-relevant bacteria in water

**Fig. 3:**
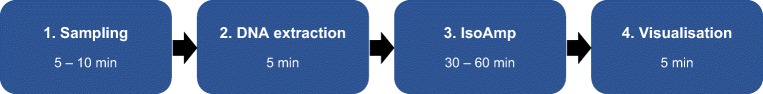
Envisioned workflow based on novel developments for rapid molecular diagnostics in resource-limited or on-site environments. With this approach, an analysis can be performed within approximately 45 to 80 min

Based on the recent developments described in the previous sections, we envision a completely on-site applicable workflow to detect SFIB in water (Fig. 3). After taking the water samples directly by employing a disposable syringe, the cells are collected on an exchangeable syringe filter. The cells are then backflushed in a much smaller volume of buffer. Subsequently, ionic liquids would be added to the eluted water sample for cell lysis and extraction of the bacterial DNA. This DNA extract can be used directly for the successive LAMP assay, which allows amplification of the target DNA sequence found in gastrointestinal *Enterococcus* species. The amplification products can be visualized by monitoring product formation in real time using a smartphone camera. If a qualitative statement on the presence of SFIB is sufficient, it would also be conceivable to add a fluorescence dye after the reaction has finished.

After elucidating these four steps, it becomes clear that isothermal DNA amplification methods per se are well established and versatile, but the associated preceding and subsequent steps still need to be adopted for simple and rapid molecular diagnostics. Similar to the case study, our laboratory has proposed another on-site applicable workflow to detect genetically modified (GM) maize in food and feed. It combines an IL-based rapid DNA extraction protocol with a newly developed HDA assay [31]. This procedure might be even further advanced by applying a DNA lateral flow test after the amplification reaction, which would allow the detection of the products by eye in 5 to 10 min without additional equipment [32]. By combining these techniques to an entire workflow, the necessary time for the analysis of GM maize is shortened from approximately 6 h to less than 2 h. Advantageously, it may be performed using only a pipette and a simple portable heating block rather than requiring a fully equipped molecular biological laboratory.

## Perspectives and novel approaches

In recent years, much work has been conducted to integrate isothermal DNA amplification methods in microfluidic devices [33, 34]. This should help avoid most of the fluid handling steps, with the potential to integrate DNA extraction, amplification, and detection on a single chip the size of a microscopy slide [33]. Microfluidic chips also allow a significant reduction in the reagent and sample volume, allowing multiplexed parallel reactions while being easy to use even for non-experts [35]. More recently, isothermal assays have also been implemented in paper microfluidic chips with wax-printed channels and reaction compartments [36].

During the manufacturing process of such microdevices, all the required reagents may be introduced in a lyophilized form. This allows the storage of the reagents without cooling for at least 100 days [12]. The extracted sample can simply be injected into a loading channel that rehydrates the dried components. This offers the advantage of increasing the investigated sample volume, thereby positively affecting the detection limit of the assay [37]. Thereafter, the reaction can be started by placing the microchip into a portable platform, which would need to be a pocket-sized device that provides the necessary reaction temperature and that includes a simple detection system without external power sources.

A major step towards making isothermal DNA amplification field applicable would be to simplify the analysis platforms—e.g., by avoiding the need for electric components that provide the thermal energy required for isothermal heating. In 2016, Liao et al. reported the development of a smartphone-sized cup containing a phase-change material that maintains a constant temperature between 60 and 65 °C for the desired duration. An exothermic reaction, which is triggered by the contact of water with an Mg-Fe alloy, provides the thermal energy in the process. Also known as flameless ration heaters, these alloys were originally developed to heat ready-to-eat meals and are therefore commercially available at low cost while ensuring high efficiency and safety [38]. Such systems would greatly benefit field testing in low-resource settings by eliminating the need for battery-powered devices. For this purpose, inexpensive reaction chambers with self-heating disposable materials could be provided along with the test kit. In 2014, Jiang et al. investigated the use of solar heating integrated into microfluidics, enabling the energy requirements for amplification reactions to be served by a smartphone battery for 70 h [39].

## Commercialized isothermal methods for food and water analysis

Various commercial products combining isothermal DNA amplification assays with portable platforms have become available in recent years. While most of them detect causative agents in clinical specimens, some platforms also employ ready-to-use test kits for applications in food and environmental analysis [24]. This includes the Loopamp Realtime Turbidimeter, an instrument for measuring the turbidity generated by the LAMP reaction in real time in combination with reagent kits to detect pathogens in foods and water (e.g., *Salmonella*, *Listeria*, *Legionella*, and *Cryptosporidium*). Another prominent example is the Genie® III system, which comes with various LAMP kits to detect key food-borne pathogens or specify food ingredients, such as meat of a large range of animals, or important crops in processed foods [40]. Additionally, the 3M™ Molecular Detection System provides a portable solution with ready-to-use LAMP kits to detect the most important food-borne pathogens. In a research article from 2015, their *Salmonella* LAMP kit was evaluated based on various food samples from Singapore [41].

In general, however, very few commercial applications of isothermal amplification methods in this area can be found compared with products based on (q)PCR or immunoassays. Not surprisingly, no food- or water-related applications have been tested by official authorities thus far with respect to low-resource settings. In this regard, clinical research is one step ahead, as exemplified by the LAMP assay for the detection of tuberculosis (TB-LAMP) for African markets. In 2013, this TB-LAMP was evaluated by an international expert panel on behalf of WHO, aiming to replace microscopy to improve the accuracy of TB detection in regions without proper laboratory infrastructure [42]. Only recently was this method tested in rural Uganda, where the authors even demonstrated improved performance in detecting TB in the sputum of patients with an HIV co-infection [43]. The effort of public authorities to implement isothermal methods as alternative diagnostic tools in developing countries shows that this technology could also be used to analyze food and water directly in the field.

## Outlook

With more than 200 annual publications in food-related isothermal amplification methods alone, one quickly loses track of the vast number of available assays. In some cases, there are even multiple different approaches to detect the very same target. Particularly, when public authorities or companies might become interested in using this technology commercially, immediate questions might be raised about which test should be used and for which application. Although many reviews summarize and discuss all isothermal DNA amplification assays considering a particular topic, these might already be outdated several months after their publication. Furthermore, much research cannot even be perceived by all interested parties, depending on their licenses for the respective publishers or journals. For these reasons, it would be highly beneficial to set up an open access database comprising a broad collection of isothermal DNA amplification assays from the areas of food, environmental, and clinical diagnostics. This database should also contain the specifications of the respective assays, such as the target analyte and corresponding matrix, oligonucleotide sequences, reaction setup, and reaction conditions. Moreover, the most important performance characteristics should be included—i.e., sensitivity, specificity, limit of detection, observed matrix effects, and the results of the analysis of real samples that have been used to further evaluate the assay.

Another issue we want to re-emphasize here is the importance of isothermal DNA amplification methods for applications in disaster areas and developing countries. Particularly, drinking water resources and basic foods must be monitored regularly also in resource-limited settings to avoid the ingestion of pathogenic microorganisms. Especially for SFIB in water, there are different thresholds that define the quality standards for drinking or bathing water, respectively. Therefore, it is particularly important for such applications that a (semi)quantitative method is available that can provide information about the degree of contamination of the sample. Although several test systems are available for the on-site detection of the most important analytes in these areas, all are based on cultivating the targeted microorganisms, often taking at least 18 h until reliable results are obtained. This testifies to the strong need for a pocket-sized device serving as a platform for interchangeable isothermal DNA amplification assays. Such a platform would require only a heating element and a sensor combined with a simple signal read-out to detect and quantify product formation. This simple setup would result in a very low price, making the platform affordable for regions without any laboratory infrastructure. The consumables would come in the form of prefabricated reaction tubes, microfluidic cartridges, or even paper strips containing all necessary reagents for DNA extraction and subsequent amplification, adapted to the analyte of interest. These target-specific components could be simply inserted into the device after the sample is applied with a disposable pipette or syringe, and the results would be available within 30 to 60 min. While most efforts still concentrate on expensive and inconvenient benchtop devices, the future focus must be on combining all technical and methodological advancements towards realizing such platforms for resource-limited settings and point-of-care applications [44].
